# A Role for H_2_O_2_ and TRPM2 in the Induction of Cell Death: Studies in KGN Cells

**DOI:** 10.3390/antiox8110518

**Published:** 2019-10-29

**Authors:** Carsten Theo Hack, Theresa Buck, Konstantin Bagnjuk, Katja Eubler, Lars Kunz, Doris Mayr, Artur Mayerhofer

**Affiliations:** 1 Biomedical Center Munich (BMC), Cell Biology, Anatomy III, Ludwig-Maximilian-University (LMU), D-82152 Planegg, Germany; theo.hack@campus.lmu.de (C.T.H.); theresa.buck@lrz.uni-muenchen.de (T.B.); Konstantin.Bagnjuk@lrz.uni-muenchen.de (K.B.); Katja.Eubler@lrz.uni-muenchen.de (K.E.); 2 Division of Neurobiology, Department Biology II, Ludwig-Maximilian-University (LMU), D-82152 Planegg, Germany; lars.kunz@biologie.uni-muenchen.de; 3 Institute of Pathology, Ludwig-Maximilian-University (LMU), D-80337 München, Germany; doris.mayr@med.uni-muenchen.de

**Keywords:** ovary, calcium channel, Trolox, granulosa cell tumor, cell death, mitochondria

## Abstract

Recent studies showed that KGN cells, derived from a human granulosa cell tumor (GCT), express NADPH oxidase 4 (NOX4), an important source of H_2_O_2_. Transient receptor potential melastatin 2 (TRPM2) channel is a Ca^2+^ permeable cation channel that can be activated by H_2_O_2_ and plays an important role in cellular functions. It is also able to promote susceptibility to cell death. We studied expression and functionality of TRPM2 in KGN cells and examined GCT tissue microarrays (TMAs) to explore in vivo relevance. We employed live cell, calcium and mitochondrial imaging, viability assays, fluorescence activated cell sorting (FACS) analysis, Western blotting and immunohistochemistry. We confirmed that KGN cells produce H_2_O_2_ and found that they express functional TRPM2. H_2_O_2_ increased intracellular Ca^2+^ levels and N-(p-Amylcinnamoyl)anthranilic acid (ACA), a TRPM2 inhibitor, blocked this action. H_2_O_2_ caused mitochondrial fragmentation and apoptotic cell death, which could be attenuated by a scavenger (Trolox). Immunohistochemistry showed parallel expression of NOX4 and TRPM2 in all 73 tumor samples examined. The results suggest that GCTs can be endowed with a system that may convey susceptibility to cell death. If so, induction of oxidative stress may be beneficial in GCT therapy. Our results also imply a therapeutic potential for TRPM2 as a drug target in GCTs.

## 1. Introduction

In a recent study, we described expression of NADPH oxidase 4 (NOX4) [1] in vivo in human granulosa cells (GCs) of ovarian follicles and in vitro in granulosa-lutein cells derived from in vitro fertilization patients. Activity of this enzyme is linked to the generation of H_2_O_2_ [2], which is a diffusible reactive oxygen species (ROS) and has been postulated to be an important signaling molecule within the follicle (e.g., [3]). Although precise modes of action remain to be identified, involvement in GC proliferation has been suggested by studies employing the granulosa cell tumor (GCT) line KGN [4] and a specific NOX4 blocker [2].

These results are in line with the changing view of ROS. They are no longer regarded as destructive correlates of oxidative stress only, but their importance in the regulation of cellular functions and in the maintenance of the essential redox homeostasis is being more and more recognized [5,6,7]. Yet, ROS in higher concentrations are indeed often associated with cell death [8,9,10].

Cellular actions of H_2_O_2_ are linked to transient receptor potential melastatin 2 (TRPM2) channel, a cation channel permeable for Ca^2+^ that is activated by oxidative stress and therefore considered to be a cellular redox sensor [11,12]. Studies in recent years have provided evidence of a role of TRPM2-mediated Ca^2+^ influx in physiological and pathophysiological functions, such as insulin release by pancreatic β-cells, pro-inflammatory cytokine production in immune cells, endothelial permeability and cell death [11]. Cell death is the most outstanding and common consequence of TRPM2 channel activation, and has been described in several publications (e.g., [8,13,14]). The exact mechanism of TRPM2 activation by H_2_O_2_ is still a subject of ongoing research; however, there are well described inhibitors such as N-(p-Amylcinnamoyl)anthranilic acid (ACA) that are widely being used in studies on TRPM2 [15,16,17].

Information about TRPM2 in ovarian cells is sparse. However, data mining of recently published single-cell RNA sequencing data has revealed that this channel is expressed in human GCs in situ [18]. To our knowledge, TRPM2 expression in GCT has not been explored yet.

In the present study we examined KGN, a model for GCT [4,19]. KGN cells express NOX4 and generate H_2_O_2_ [2]. We found that they also express TRPM2, which can be activated by H_2_O_2_ and facilitate an influx of Ca^2+^, followed by mitochondrial fragmentation and cell death. Immunohistochemical analysis of tissue microarrays (TMAs) revealed that both NOX4 and TRPM2 were expressed by all GCT samples we examined. Our findings suggest that induction of oxidative stress in GCT may result in cell death. Furthermore, the results implicate a therapeutic potential of TRPM2 as a possible drug target.

## 2. Materials and Methods

### 2.1. KGN Cell Culture

Procedures have recently been described [2]. The patented KGN cell line was obtained from RIKEN BioResource Center [4] with permission by T. Yanase. KGN cells were cultured in Dulbecco’s Modified Eagle’s Medium (DMEM)/Ham’s F12 medium (Life Technologies, Paisley, UK) supplemented with penicillin (100 U/mL), streptomycin (100 μg/mL) (Biochrom, Berlin, Germany) and 10% fetal calf serum (FCS) (Capricorn Scientific, Ebsdorfergrund, Germany) at 37 °C and with 5% CO_2_. For stimulation experiments, 20 µM Trolox (Santa Cruz Biotechnology, Dallas, TX, USA) or 100 µM/1 mM H_2_O_2_ (Sigma-Aldrich, St. Louis, MO, USA) was diluted in DMEM/Ham’s F12 medium (Life Technologies; colorless medium without phenol red was used for live cell fluorescence imaging to reduce background autofluorescence).

### 2.2. Reverse Transcription PCR

RNeasy Plus Micro Kit (Qiagen, Hilden, Germany) was used to isolate RNA. Concentration and purity were measured as described [2]. Superscript II (Invitrogen, Carlsbad, CA, USA) and random 15-mer primers (metabion international, Munich, Germany) were used for reverse transcription (RT). Oligonucleotide primers for amplification of NOX4 were described previously [2]. For TRPM2, we used primers with the following sequences: 5′–AGGCTGAACTCTAACCTGCAC–3′ (forward) and 5′–GGAGGAGGGTCTTGTGGTTC–3′ (reverse) (yielding a 103 bp fragment). Negative controls were performed by replacing cDNA with RNA (-RT) or water (H_2_O). Amplicon identity was verified by agarose gel electrophoresis, consecutive cDNA extraction with Wizard SV Gel and PCR Clean-up System (Promega, Madison, WI, USA) and sequence analysis (GATC, Konstanz, Germany).

### 2.3. Western Blotting

Protein isolation and Western blotting were performed as previously described [20]. KGN cells were lysed using RIPA buffer plus protease and phosphatase inhibitors (Thermo Fisher Scientific, Waltham, USA). A total of 7 µg (NOX4) or 20 µg (cleaved caspase 3, clCASP3) protein per lane was loaded on a 10% (NOX4) or 12% (clCASP3) SDS gel and run (NOX4: 20 min at 100 V + 70 min at 120 V; clCASP3: 20 min at 75 V + 40 min at 150 V). After blotting (NOX4: 55 min at 100 V; clCASP3: 60 min at 100 V) and blocking with 5% non-fat dry milk (Roth, Karlsruhe, Germany) in Tris-buffered saline with Tween 20 (5 mM Tris, 100 mM NaCl, 0.05% Tween 20, pH 7.5), rabbit anti-NOX4 polyclonal antiserum (1:1000, #7927, ProSci, Fort Collins, CO, USA) or rabbit anti-clCASP3 monoclonal antibody (1:1000, #9664, Cell Signaling Technology, Danvers, MA, USA) were administered to detect these proteins. As a loading control, mouse anti-β-actin monoclonal antibody (1:10000, #A5441, Sigma-Aldrich) was used. HRP-conjugated goat anti-rabbit and goat anti-mouse secondary antibodies (Jackson ImmunoResearch Europe, Cambridgeshire, UK) were used to visualize specific binding. Band intensities were determined using the FIJI software [21].

### 2.4. Immunohistochemistry

Immunohistochemistry was performed using TMAs assembled from anonymized archival material. All patients were treated surgically at the same institution (Department of Gynaecology, University of Munich, Germany) and diagnosed at the Institute for Pathology, LMU, Munich, Germany. The diagnoses were confirmed by an experienced gynaecopathologist (D.M.). Tissue biopsies (*n* = 73) were taken from representative regions of larger paraffin-embedded tumor samples and arrayed into a new recipient paraffin block using MTA-1 (Manual Tissue Arrayer) from Beecher Instruments, Sun Prairie, WI, USA. Staining procedures were conducted as previously described [22]. In brief, sections were deparaffinized, antigens were unmasked and endogenous peroxidase activity was blocked, followed by incubation in 10% goat serum, diluted in PBS, to prevent unspecific binding. Polyclonal rabbit antisera raised against human NOX4 (1:500, #7927, ProSci, Fort Collins, CO, USA) or against human TRPM2 (1:100, #HPA035260, Sigma-Aldrich) were used to identify these proteins in the TMAs. Specific binding was detected by biotinylated goat anti-rabbit secondary antibody and Vectastain Elite ABC kit (Vector Laboratories, Burlingame, CA, USA). For negative controls, incubation with normal rabbit serum instead of the primary antiserum was performed. Sections were counterstained with haematoxylin and visualized using a Zeiss Axioplan microscope with the Achroplan 63x/0.80 objective (Carl Zeiss Microscopy, Jena, Germany) and a Jenoptik camera (Progres Gryphax Arktur; Jenoptik, Jena, Germany).

### 2.5. Measurement of H_2_O_2_

The generation of H_2_O_2_ was measured using an Amplex Red Hydrogen Peroxide/Peroxidase Assay Kit (Life Technologies, Eugene, OR, USA) as described previously [2,23]. In brief, KGN cells were seeded in black 96-well plates (1.5 × 10^4^ cells/well, *n* = 6) and cultured overnight. Amplex Red reagent (10-acetyl-3,7-dihydroxyphenoxazine) was used in a final concentration of 5.0 µM and fluorescence levels were measured at 544 nm excitation/590 nm emission in a fluorometer (FLUOstar Omega, BMG LABTECH, Ortenberg, Germany) for 115 min at 37 °C. Data points were normalized according to the starting point value. To compare H_2_O_2_ concentrations in the supernatant, 3 × 10^5^ cells were seeded on a 60-mm (diameter) cell culture dish the day before stimulation. After 72 h of stimulation with 20 µM Trolox or serum-free medium only, supernatants were collected, centrifuged and measured with the Amplex Red Kit according to the manufacturer’s instructions (*n* = 3).

### 2.6. Cell Viability Assay, Confluence Measurement and Cell Counting

Cell viability was estimated by measuring cellular ATP content (the indicator for metabolically active cells) using CellTiter-Glo Luminescent Cell Viability Assay (Promega, Madison, WI, USA) as described previously [2,24]. KGN cells were seeded on a white 96-well microtiter plate (5.0 × 10^3^ cells/well, *n* = 12) one day prior to stimulation, then exposed to 20 µM Trolox or serum-free medium for 72 h. After removal of the supernatant and washing with PBS, wells were filled with a 1:1 mixture of CellTiter-Glo reagent and DMEM/Ham’s F12 without phenol red (100 µl/well), mixed on a plate shaker and incubated for 10 min at room temperature. Luminescence was measured by a luminometer (FLUOstar Omega; BMG LABTECH). Confluence was analyzed with the JuLIBr Live cell movie analyzer (NanoEnTek, Waltham, MA, USA) for a period of 72 h. For determination of cell numbers, KGN cells were counted using the CASY Cell Counter (OLS OMNI Life Science, Bremen, Germany).

### 2.7. Fluorescence-Activated Cell Sorting (FACS) Analysis

FITC-conjugated annexin V (ALX-209-256-T100, Enzo Life Sciences, Farmingdale, NY, USA) and SYTOX Red Dead Cell Stain (S34859, Invitrogen) were used to examine the occurrence of apoptosis. KGN cells were incubated in colorless DMEM/Ham’s F12 medium for 24 h with or without 100 µM H_2_O_2_, or for 72 h with or without 20 µM Trolox. They were trypsinized, washed with PBS and incubated with annexin V-FITC (2.5 µg/mL) according to the manufacturer’s instructions. SYTOX Red Dead Cell Stain—a nucleic acid stain labeling cells with damaged membranes—was added (1:2000), and cells were analyzed using the BD FACSCanto (Becton, Dickinson and Company, Franklin Lakes, NJ, USA). Annexin V-FITC signal was obtained using a 488 nm excitation laser and a 530/30 bandpass (BP) filter. For the SYTOX Red signal, a 633-nm laser and a 660/20 BP filter were used, with 20,000 events recorded for each treatment. Signals were analyzed using BD FACSDiva Software (version 8.0.1, Becton, Dickinson and Company). Cells show single staining with annexin V in an early stage of apoptosis (intact membrane), while they are double-positive for annexin V and nucleic acid stains in late apoptosis (cf. [25,26]). The apoptotic/late apoptotic (L/A) fraction of the analyzed cells comprise early and late stage apoptosis.

### 2.8. Calcium Imaging

For all calcium imaging experiments, KGN cells were incubated with 5 µM Fluoforte Reagent (Enzo Life Sciences)—a fluorescent dye detecting intracellular Ca^2+^—in DMEM/Ham’s F12 without phenol red for 30 min at 37 °C and 5% CO_2_ in ibidi dishes optimized for microscopy (µ-dish 35 mm, ibidi, Gräfelfing, Germany). After washing with colorless medium, fluorescence was monitored every 5 s using a wide-field microscope (microscope: Axio Observer.Z1; light-source: Colibri.2; camera: Axiocam 506 mono; objective: Plan-Apochromat 20x/0.8 Ph2 M27; software: ZEN 2.6; Carl Zeiss Microscopy) with a 450–490 nm BP excitation and 500–550 nm BP emission filter (F46-002; AHF analysentechnik, Tübingen, Deutschland). A continuous flow of medium with or without stimulant was generated by a peristaltic pump (Instech Laboratories, Plymouth Meeting, PA, USA) linked to needles that were placed under the surface of the medium, close to the observed cells. H_2_O_2_ was utilized in a higher concentration (1 mM) to address diluting effects. For the blocking experiments, KGN cells were incubated with either ACA (20 µM, Sigma-Aldrich) or DMSO (solvent control) for 4 h prior to and during measurements. Stimulation with 0.05‰ trypsin (Biochrom, Berlin, Germany) served as a positive control (cf. [27,28]). FIJI software was used to obtain fluorescence intensities of the regions of interest (ROIs) and to optimize the images and videos provided in the Supplementary Materials. Background fluorescence was subtracted from the raw data and results were normalized to the starting point values. Images and videos showed fluorescence intensity based on a pseudo-color scale from black/red (low Ca^2+^) to yellow/white (high Ca^2+^).

### 2.9. Mitochondrial Imaging

KGN cells were incubated with 100 nM MitoTracker Green FM (Molecular Probes, Eugene, OR, USA) for 30 min at 37 °C and 5% CO_2_ in microscopy-optimized cell culture dishes (µ-dish 35 mm, ibidi). The staining solution was prepared in DMEM/Ham’s F12 without phenol red. To examine changes of the mitochondrial structure, cells were treated with 100 µM H_2_O_2_ after staining and washing. Fluorescence was recorded with a wide-field microscope (microscope: Axio Observer.Z1; light-source: Colibri.2; camera: Axiocam 506 mono; objective: Plan-Apochromat 63x/1.40 Oil Ph 3 M27; software: ZEN 2.6; Carl Zeiss Microscopy). A 450–490 nm BP (excitation) and a 500–550 nm BP (emission) were used (F46-002; AHF analysentechnik). In a second approach, stimulation with H_2_O_2_ for 4 h in colorless medium prior to staining and imaging was performed to rule out phototoxicity due to multiple imaging as a reason for mitochondrial fragmentation.

To evaluate the effect of H_2_O_2_ (100 µM) on mitochondria, 239 control cells and 122 treated cells in two dishes each for both groups were analyzed after 4 h of treatment. Examples for KGN cells with elongatedor fragmented mitochondrial networks are given in the corresponding figure.

### 2.10. Statistics

GraphPad Prism 6.0 Software (GraphPad Software, San Diego, CA, USA) was used to perform unpaired *t*-tests (two-tailed) for comparisons of H_2_O_2_, ATP and cell counts. Control and Trolox-treatments were performed in parallel (*n* = 3), derived samples were run next to each other on the gels and results (band intensities) of clCASP3 Western blots were analyzed using paired *t*-test.

## 3. Results

### 3.1. KGN Cells Express H_2_O_2_ Generating NOX4

**Figure 1:**
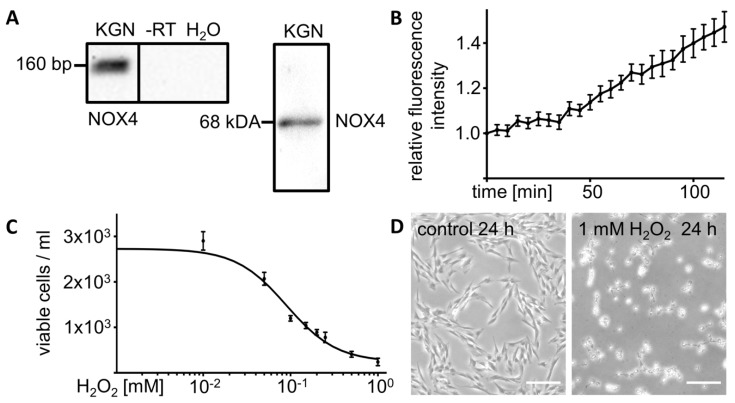
H_2_O_2_ production and release by cultured KGN cells and effects of exogenous H_2_O_2_. (**A**) NOX4 RT-PCR analysis and Western blot of cultured KGN cells show single bands of 160 bp and 68 kDa, respectively. Controls using RNA (-RT) or H_2_O instead of cDNA (H_2_O) were negative. (**B**) Hydrogen peroxide assay of untreated KGN cells showed increasing H_2_O_2_ levels in the supernatant over a time period of 2 h (*n* = 6). Signal intensities were normalized to start point values. Bars indicate SEM. (**C**) Exogenously added H_2_O_2_ reduced cell viability in a dose dependent manner. Cell counts after treatment of KGN cells with different concentrations of H_2_O_2_ for 24 h (*n* = 2–5 for each concentration) are shown with an interpolated sigmoidal standard curve (*r^2^* = 0.9361). Bars indicate SEM. (**D**) Images of KGN cells treated with H_2_O_2_ (1 mM) for 24 h compared to untreated control cells. Scale bars indicate 200 µm.

Expression of NOX4, an enzyme known to generate H_2_O_2_, was detected by Western blot (68 kDa) and RT-PCR (160 bp) (Figure 1A). The Amplex Red Hydrogen Peroxide Assay (*n* = 6) provided evidence for basal H_2_O_2_ production and release by untreated KGN cells, resulting in increasing levels in the supernatant over time (Figure 1B).

### 3.2. Exogenous H_2_O_2_ Kills KGN Cells in a Dose Dependent Manner

To examine effects of H_2_O_2_, KGN cells were exposed to H_2_O_2_ at different concentrations for 24 h (*n* = 2 to 5 for each concentration). We observed a dose dependent reduction of cell viability (cell counting, live cell imaging). Cell numbers decreased with a calculated EC_50_ of 89.7 µM (72.16–111.5 µM; interpolated sigmoidal standard curve:$\;f\left(x\right)=23800+\frac{272929-23800}{1+10^{\left(1.953-x\right)\left(-1.507\right)}}$; *r^2^* = 0.9361) (Figure 1C). Figure 1D shows a live cell image of KGN cells treated with H_2_O_2_ (1 mM) for 24 h compared to controls, revealing the damaging effects of exogenous H_2_O_2_.

### 3.3. Trolox Promotes Survival of KGN Cells in Serum-Free Medium

**Figure 2:**
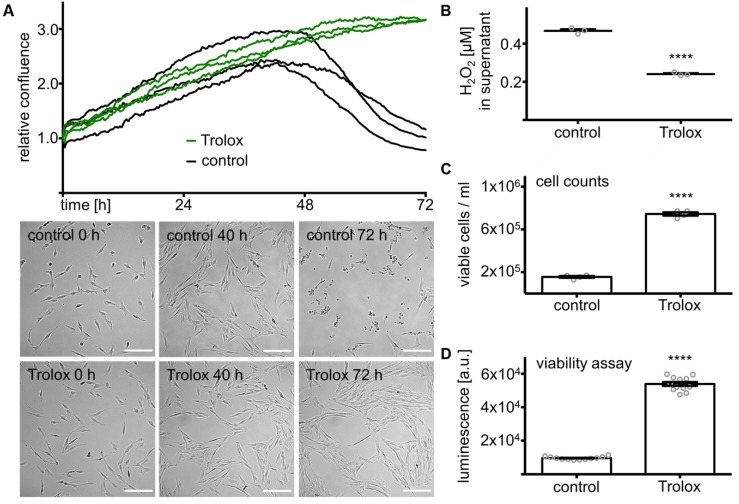
Antioxidant Trolox antagonized the effects of endogenous H_2_O_2_. (**A**) Changes in confluence of KGN cells over the course of 72 h for cells treated with Trolox (20 µM) compared to serum-free medium only (*n* = 3). Results were normalized to the respective start value. Images at different time points are shown below. Scale bars indicate 200 µm. (**B**) H_2_O_2_ in the supernatant after treatment with Trolox (20 µM) for 72 h, measured by a hydrogen peroxide assay, was significantly lower compared to ontrols (*n* = 3, **** *p* < 0.0001). Means and SEM, as well as individual results are given. (**C**) Cell counts relative to the average untreated controls significantly increased (*n* = 4, **** *p* < 0.0001). Means and SEM, as well as individual results are shown. (**D**) ATP viability assay-generated luminescence signaling was significantly higher in Trolox-treated cells (*n* = 12, **** *p* < 0.0001) after 72 h of treatment. Means and SEM, as well as individual results (circles), are presented.

Culturing KGN cells in serum-free medium for 72 h led to a drop in confluence after an initial 2.6 ± 0.2 fold increase for the first 40 h (mean). This decrease was prevented by the addition of Trolox (20 µM), a water-soluble derivate of vitamin E. Trolox is well known for its antioxidative activity [29,30] and kept KGN cells prospering until the end of the measurement. Pictures taken by a live cell imaging system show the difference between treated and control cells. While KGN cells looked healthy within the first part of the observation under both conditions, they detached after 72 h under control conditions, but were further propagated with Trolox (Figure 2A). The H_2_O_2_ concentration in the supernatant of KGN cells after 72 h in serum-free medium was significantly (*n* = 3, *p* < 0.0001, *t*-test) reduced by Trolox (Figure 2B). Cell counts (*n* = 4, *p* < 0.0001, *t*-test) (Figure 2C) and viability assay (*n* = 12, *p* < 0.0001, *t*-test) (Figure 2D) after 72 h gave further evidence for the positive effects of Trolox on KGN cell survival in serum-free medium.

### 3.4. Effects of Cultivation in Serum-Free Medium and Exogenous H_2_O_2_ on Markers of Apoptosis and Necrosis

**Figure 3:**
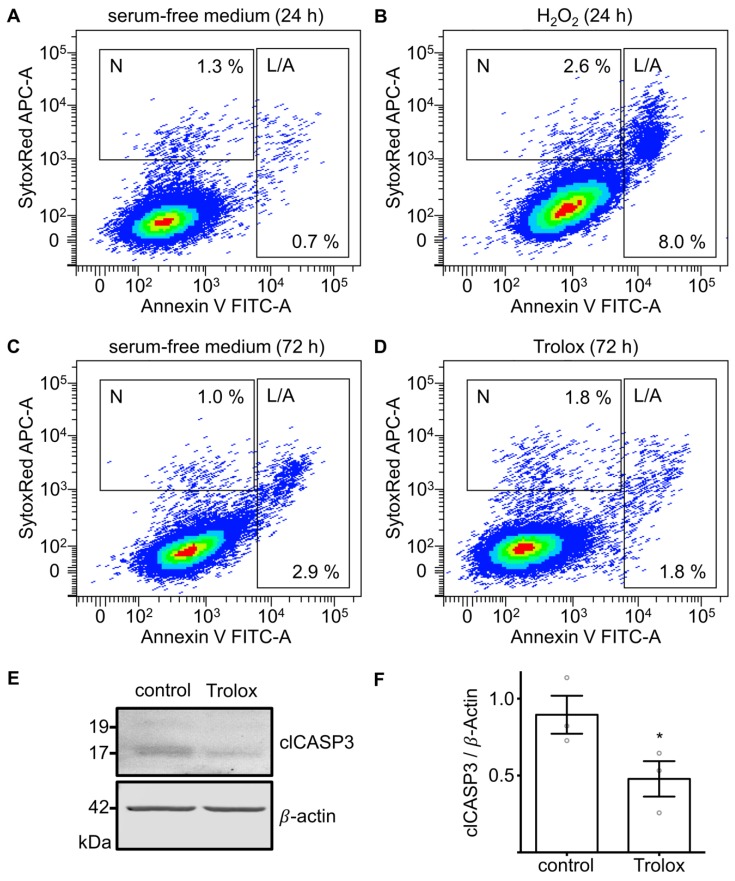
Effects of serum-free medium, exogenous H_2_O_2_ and Trolox on markers of apoptosis and necrosis. (**A**–**D**) FACS analysis of KGN cells co-stained with annexin V and SYTOX Red Dead Cell Stain. N indicates necrotic (single stained with SYTOX Red), apoptotic and late apoptotic (L/A) cells (single stained with annexin V or double-stained). Unstained cells in the left lower quadrant were viable. Percentage values of the N and L/A fractions are shown for each treatment: (**A**) Serum-free medium for 24 h; (**B**) H_2_O_2_ (100 µM) in serum-free medium for 24 h; (**C**) serum-free medium for 72 h; (**D**) Trolox (20 µM) in serum-free medium for 72 h; (**E**) Western blot membrane with clCASP3 and β-actin bands; (**F**) clCASP3 levels in KGN cells after treatment with Trolox (20 µM) for 72 h are compared with control cells in serum-free medium. clCASP3 relative to β-actin was significantly lower in treated cells (*n* = 3, * *p* < 0.05). Means and SEM and individual results are shown.

FACS analysis of KGN cells co-stained with FITC-conjugated annexin V and the nucleic acid stain SYTOX Red Dead Cell Stain revealed an 11.4-fold increase in the apoptotic/late apoptotic (L/A) fraction, while the necrotic fraction (N) only doubled in cells treated with H_2_O_2_ (100 µM) for 24 h compared to the serum-free medium control (Figure 3A,B). Culturing KGN cells in serum-free medium resulted in a 4.1-fold higher L/A cell fraction after 72 h compared to 24 h, whereas the N fraction reduced by 23% (Figure 3A,C).

### 3.5. Treatment with Trolox Reduces Markers of Apoptosis

Addition of Trolox (20 µM) to the serum-free medium reduced the L/A fraction after 72 h in serum-free medium by 37.9% (Figure 3C,D), but not the N fraction, which actually increased. Western blot analysis confirmed the effect on apoptosis by showing a significant reduction in clCASP3 (*n* = 3, *p* = 0.0225, paired *t*-test) (Figure 3E,F).

### 3.6. KGN Cells Express Functional TRPM2

**Figure 4:**
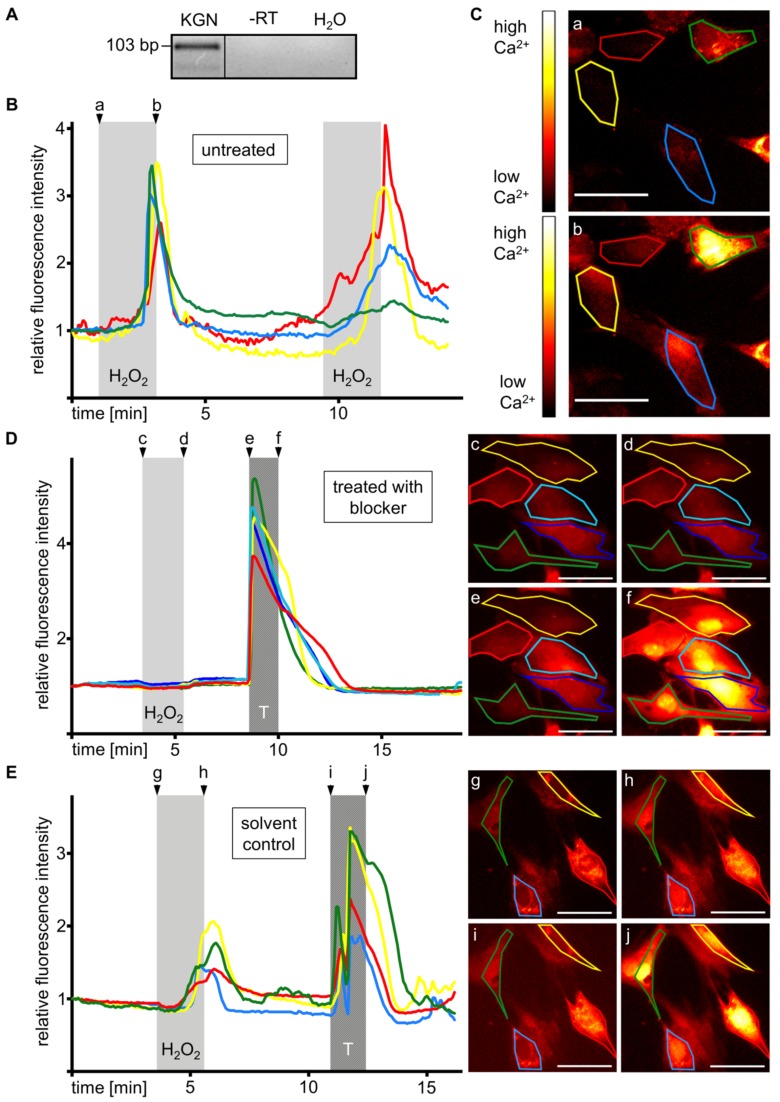
KGN cells express functional TRPM2. (**A**) TRPM2 RT-PCR shows a band at 103 bp. Controls with RNA (-RT) or H_2_O instead of cDNA (H_2_O) were negative. (**B**) Addition of H_2_O_2_ (1 mM) increased the fluorescence signal of the four individual KGN cells shown, which were loaded with the Ca^2+^-sensitive dye Fluoforte. Background signals were subtracted and fluorescence is shown relative to the respective start value of each region of interest (ROI). (**C**) Fluorescence images, taken before (**a**) and after (**b**) the first stimulation with H_2_O_2_. (**D**) Treatment with the inhibitor (ACA; 20 µM), 4 h prior to and during the measurement, blocked the Ca^2+^ increase upon stimulation with H_2_O_2_, but not with 0.05‰ trypsin (T). Images (**c**–**f**) represent the indicated time points. (**E**) The H_2_O_2_-derived Ca^2+^ increase was obtained in the DMSO control and thus ruled out solvent effects. Images (**g**–**j**) represent the indicated time points. The pseudo-color scale shown in (**c**) applies for all live cell images. Colored frames mark the cells represented in the corresponding graphs. Scale bars indicate 50 µm.

Expression of TRPM2 channel, a H_2_O_2_-responsive Ca^2+^-permeable cation channel, was detected by RT-PCR (Figure 4A) and sequencing. To examine functionality of TRPM2, changes in intracellular Ca^2+^ levels were imaged. Stimulation with H_2_O_2_ (1 mM) caused a transient increase in Ca^2+^ levels in three independent measurements, which occurred with a delay of more than 1 min and quickly disappeared after terminating the stimulation. Repeated stimulation was possible (Figure 4B,C). The Ca^2+^ increase was blocked by treatment with the TRPM2 inhibitor ACA (20 µM) [15,16,17] applied for 4 h prior to and during the measurement. Cellular response to the positive control (trypsin) was not affected (Figure 4D). The H_2_O_2_-derived signal was obtained in the control experiments with the solvent (Figure 4E). Videos are provided in the Supplementary Materials (Videos S1–S3). Blocking experiments and according controls were repeated in four independent measurements each.

### 3.7. Exogeneous H_2_O_2_ Causes Mitochondrial Fragmentation

**Figure 5:**
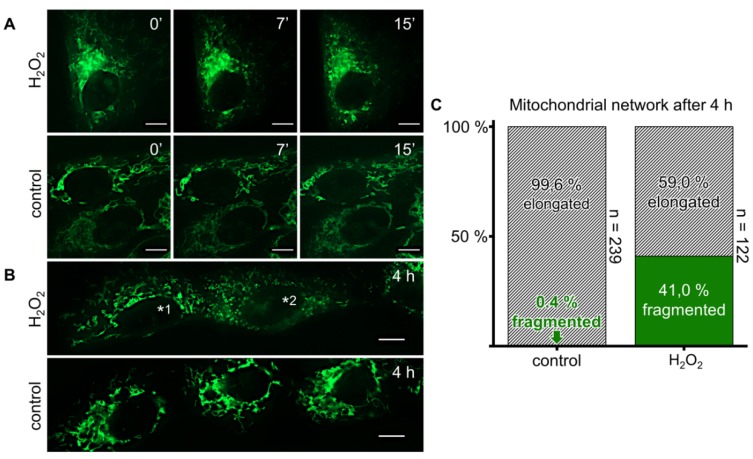
H_2_O_2_ causes mitochondrial fragmentation. (**A**) MitoTracker Green FM-based live cell fluorescence images show mitochondria of KGN cells treated with H_2_O_2_ (100 µM) at different time points, compared to medium-only controls. (**B**) KGN cells stained and imaged after 4 h of treatment with H_2_O_2_ (100 µM) or medium only. ^*1^ indicates a cell presenting an elongated mitochondrial network, ^*2^ indicates an example of a fragmented mitochondrion. (**C**) Portion of cells with fragmented mitochondria after 4 h, as determined by counting 239 control and 122 treated KGN cells. Mitochondrial fragmentation was markedly increased in H_2_O_2_-treated cells. Scale bars (**A**,**B**) indicate 10 µm.

Monitoring mitochondria of KGN cells during treatment with H_2_O_2_ (100 µM) revealed fragmentation over time (Figure 5A). Stimulation for 4 h prior to staining and imaging was performed to rule out phototoxicity due to multiple imaging during time series as a reason for fragmentation. Mitochondria were fragmented in this approach (Figure 5B), and comparison of H_2_O_2_-treated cells to the control cells revealed a vast difference in the portion of KGN cells presenting mitochondrial fragmentation. One out of 239 (0.4%) untreated and 50 out of 122 (41.0%) treated cells showed fragmentation (Figure 5C).

### 3.8. Primary GCT Express NOX4 and TRPM2

**Figure 6:**
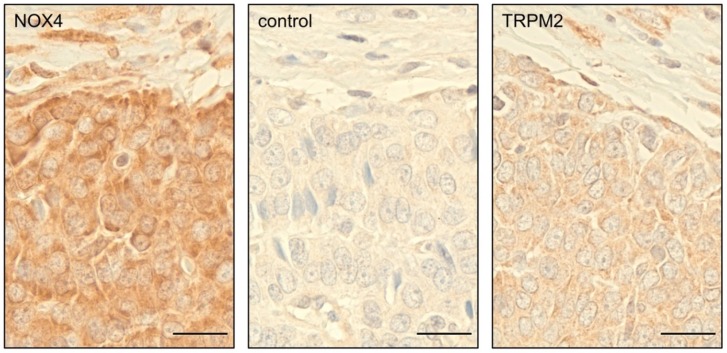
Granulosa cell tumors (GCTs) express NOX4 and TRPM2. Immunohistochemical staining of NOX4 and TRPM2 in one of the 73 tumor samples analyzed. NOX4 and TRPM2 were detected in GCT cells, normal rabbit serum control was negative. Scale bars indicate 20 µm.

As the KGN cell line serves as a well-established in vitro model for GCTs, we analyzed NOX4 and TRPM2 expression in 73 GCT samples using TMAs. Immunohistochemical analysis revealed that all of the tumors expressed both NOX4 and TRPM2 (Figure 6). Both proteins were detected in GCT cells and showed a generally homogenous distribution, but intensities varied between different tumors.

## 4. Discussion

Our results show that H_2_O_2_ from both exogenous and endogenous sources is able to induce cell death in KGN cells. The majority of endogenous H_2_O_2_ is most likely generated by NOX4, as previously shown [2]. NOX4 expression in KGN cells was confirmed by RT-PCR and Western blot. Trolox, a typical ROS scavenging antioxidant [29,30], reduced endogenously produced H_2_O_2_ in the supernatant and rescued the cells, as shown by confluence measurements, cell counting and ATP cell viability assay.

There is ample evidence implying that H_2_O_2_ activates the ROS-gated cation channel TRPM2, and that the consecutive Ca^2+^ influx may cause cell death (e.g., [8,9,10]). Single cell RNA sequencing data in a recent publication [18] revealed expression of TRPM2 in GCs of human follicles in situ. We showed that TRPM2 is expressed in the GCT-derived tumor cell line KGN and calcium imaging suggested its functionality. Intracellular Ca^2+^ levels increased upon H_2_O_2_ stimulation and disappeared after termination of the treatment. The observed delay of more than 1 min provided an additional indication of TRPM2 involvement, since its activation by H_2_O_2_ is reported to be slow and take up to minutes [11].

To provide further evidence for the functionality of TRPM2 in KGN cells, we utilized ACA to inhibit TRPM2 in the calcium imaging experiment. ACA completely blocked the Ca^2+^ influx upon H_2_O_2_ stimulation, pinpointing TRPM2 as the channel responsible for the signal observed in untreated cells. As we found in our experiments, extracellular application of ACA was reported previously to completely block the H_2_O_2_-induced increase of intracellular Ca^2+^ in TPRM2-expressing cells at a concentration of 20 µM [15]. Yet, ACA is also a phospholipase A_2_ (PLA_2_) blocker. Although the inhibitory action on TRPM2 was reported to be independent of effects on PLA_2_ [15], detrimental consequences of PLA_2_ inhibition cannot be ruled out, especially in long-term treatments. We therefore did not perform additional experiments.

In accordance with previous studies reporting apoptosis upon H_2_O_2_ stimulation in primary GCs [31] and other cells [8,9,10], we detected a distinct induction of apoptosis/late apoptosis by H_2_O_2_ in KGN cells. Although annexin V and nucleic acid stain double-stained cells are often referred to as late apoptotic [25,26], necrotic cells might be double-positive as well [32]. Given that changes in the necrotic cell fraction do not match the changes in the annexin V signals, our results provide evidence that exogenous H_2_O_2_ induces apoptosis, although other cell death forms might be involved as well.

Fetal calf serum (FCS) was reported to feature a total anti-oxidant capacity (TAC) equaling 360 ± 40 µM Trolox [33], which implicates a minor role of endogenously produced H_2_O_2_ in media supplemented with FCS. Even 10% of FCS, as usual in our culture medium, exceeds the TAC of 20 µM Trolox. For different experiments we therefore used serum-free medium. Under these conditions, H_2_O_2_ produced by cells may reach concentrations that might be high enough to activate TRPM2, especially in their immediate surroundings, although the overall concentration within the supernatant remains relatively low compared to the concentration used for exogenous stimulation with H_2_O_2_. In accordance, results of the FACS analysis and the clCASP3 Western blot revealed an induction of apoptosis by long-term cultivation in serum-free medium. Reduction of markers for apoptosis by the ROS scavenger Trolox and the differences in H_2_O_2_ levels in the supernatants suggest that endogenous H_2_O_2_ might play an important role in the induction of apoptosis. These results are in line with the ability of another antioxidant, N-acetyl-L-cysteine, to reduce H_2_O_2_-induced apoptotic cell death in human melanocytes [8].

In their review on mitochondrial dynamics and apoptosis, Suen et al. [34] discussed the role of mitochondrial fragmentation and pointed out that while there are many different conditions wherein this can be observed, it is always involved in apoptosis and appears before caspase activation. To further examine the mechanism underlying the H_2_O_2_ effects on KGN cells, we performed live cell imaging of mitochondria labeled with MitoTracker Green FM. We observed rapid and massive fragmentation of these organelles upon H_2_O_2_ stimulation. This may reflect another manifestation of an activated apoptosis machinery and be a consequence of Ca^2+^-dependent phosphorylation of dynamin-related protein 1 (Drp1) [35], a critical player in mitochondrial fragmentation [36]. The exact mechanisms in KGN cells and the question of whether other forms of cell death are involved remain to be studied.

To explore in vivo the relevance of our cellular results, we studied the expression of NOX4, a typical source for endogenous H_2_O_2_ [1,2], and TRPM2, a possible target for H_2_O_2_ [11,12], in primary GCT. Immunohistochemical analysis of TMAs revealed that both NOX4 and TRPM2 were expressed in all 73 tumors analyzed in this study. We noticed that signal intensities varied between the different GCTs. However, as we studied archival material, we reasoned that variations in sample preparations or storage could not be ruled out. Therefore, we did not attempt to further evaluate these differences.

Investigations of other tumor’s entities indicated that NOX4-derived ROS may limit tumor progression (liver carcinoma [37]) and that TRPM2 overexpression may enhance induction of cell death by H_2_O_2_ (neuroblastoma [14]). TRPM2 confers susceptibility of different tumors to H_2_O_2_-mediated neutrophil cytotoxicity, thereby limiting metastasis [38]. Interestingly, inflammatory neutrophil infiltration is mediated by TRPM2 activation, resulting in chemokine production by monocytes [39].

In line with these observations, our immunohistochemical analysis of 73 GCTs and the results of experiments with KGN cells implicate that GCTs can be endowed with a relevant system that may convey susceptibility to cell death. Our in vitro-studies provide evidence that induction of oxidative stress may be beneficial in GCT therapy and that there is a therapeutic potential for TRPM2 as a drug target. Whether the new insights of our study are indeed of relevance in vivo remains to be shown.

## Supplementary Materials

The following are available online at https://www.mdpi.com/2076-3921/8/11/518/s1: Video S1. Increasing intracellular Ca^2+^ levels upon stimulation with H_2_O_2_. Fluorescence images of the experiment represented in Figure 4B,C were compiled to a time-lapse video. The movie shows reversible increases in intracellular Ca^2+^ levels after addition of 1 mM H_2_O_2_ to KGN cells. Video S2. Time-lapse movie of the experiment represented in Figure 4D. This video shows levels of intracellular Ca^2+^ in cells treated with the TRPM2 blocker ACA. Ca^2+^ levels do not increase upon stimulation with 1 mM H_2_O_2_; yet, they increase upon stimulation with trypsin. Video S3. Obtained H_2_O_2_ effect in the solvent control. Time-lapse video of the experiment represented in Figure 4E. The movie shows that the Ca^2+^ response upon stimulation with H_2_O_2_ is obtained in the DMSO control. The second increase in Ca^2+^ derives from stimulation with trypsin.
